# Bioactive Antimicrobial Peptides: A New Weapon to Counteract Zoonosis

**DOI:** 10.3390/microorganisms10081591

**Published:** 2022-08-07

**Authors:** Luisa Zupin, Carlos André dos Santos-Silva, Aya R. Hamad Al Mughrbi, Livia Maria Batista Vilela, Ana Maria Benko-Iseppon, Sergio Crovella

**Affiliations:** 1 Institute for Maternal and Child Health—IRCCS Burlo Garofolo, 34137 Trieste, Italy; 2 College of Dental Medicine, Qatar University, Doha 2713, Qatar; 3 Centro de Biociências, Departamento de Genética, Universidade Federal de Pernambuco, Recife 50670-420, Brazil; 4 Biological Science Program, Department of Biological and Environmental Sciences, College of Arts and Sciences, Qatar University, Doha 2713, Qatar

**Keywords:** zoonosis, antimicrobial peptides, antimicrobial treatment, infection

## Abstract

Zoonoses have recently become the center of attention of the general population and scientific community. Notably, more than 30 new human pathogens have been identified in the last 30 years, 75% of which can be classified as zoonosis. The complete eradication of such types of infections is far out of reach, considering the limited understanding of animal determinants in zoonoses and their causes of emergence. Therefore, efforts must be doubled in examining the spread, persistence, and pathogenicity of zoonosis and studying possible clinical interventions and antimicrobial drug development. The search for antimicrobial bioactive compounds has assumed great emphasis, considering the emergence of multi-drug-resistant microorganisms. Among the biomolecules of emerging scientific interest are antimicrobial peptides (AMPs), potent biomolecules that can potentially act as important weapons against infectious diseases. Moreover, synthetic AMPs are easily tailored (bioinformatically) to target specific features of the pathogens to hijack, inducing no or very low resistance. Although very promising, previous studies on SAMPs’ efficacy are still at their early stages. Indeed, further studies and better characterization on their mechanism of action with in vitro and in vivo assays are needed so as to proceed to their clinical application on human beings.

## 1. Introduction

Zoonoses have recently become the center of attention of the general population and scientific community, given the emergence of the coronavirus disease 2019 pandemic (COVID-19) and the ongoing debate regarding the origin of SARS-CoV-2 from bats and its transmission to intermediate animal hosts, which has allowed the spillover of the virus to humans [1].

A zoonosis, according to the WHO definition, “is any infection transmitted from vertebrate animals to human beings, involving pathogen that may be bacterial, fungal, viral, or parasitic” [2]; the infection can be transmitted: (1) by direct exposure with infected animals (contact with body fluids through bite or scratch or touching), (2) by indirect contact with areas contaminated by infected animals, (3) vector-borne through insect bite (mainly by mosquitos and ticks), and (4) food- or water-borne contracted by eating contaminated food or water [3].

Zoonosis can be classified into three groups: endemic zoonoses, present in many areas affecting several human beings and animals; epidemic zoonoses, sporadic in time and spatial distribution; and emerging and re-emerging zoonoses, newly occurring or previously known and rapidly incrementing [4].

Emerging zoonosis accounts for 60% of the emerging infectious disease (EID) worldwide, causing annually 1 billion cases of disease and millions of deaths. Notably, more than 30 new human pathogens have been identified in the last 30 years, 75% of which can be classified as zoonosis (derived from animals) [4]. 

Zoonosis should be considered globally threatening for imposing unprecedented negative impacts on human health, society, and economy. Although different regions can be potential hotspots where many zoonoses have risen, their effects are still far-reaching since they can easily travel around the world by means of interconnection and globalization. Tropical regions present a high risk of EID, according to the EcoHealth Alliance publication [5]. Evergreen broadleaf trees, human population density, climate change, and loss of biodiversity are the factors that highly influence the EID distribution. Europe, USA, and Japan are the regions with higher probability of observing new EID, since in these territories, the surveillance monitoring is meticulously organized. In contrast, tropical regions of North America, Southeast Asia, Central Africa, and South America (Brazil) are the areas presenting a high risk of EID occurrence [5].

Considerably, humans were able to coexist with both domestic and wild animals during ancient times and suffered no great consequences. However, with the current presence of anthropogenic elements, human–animal interactions have increased intensively, putting humanity at an inevitable fate of zoonotic diseases, driven by population growth, unfeasible utilization of natural resources, and wildlife meat consumption [6]. In regions of low income, such as sub-Saharan Africa, greater than 25% of disability-adjusted life years (DALYs) were lost due to emerging zoonotic diseases. Specifically, in East Africa, endemic zoonotic diseases were found to be highly prevalent, considering the large human population that inhabits areas of livestock and wildlife [7].

The urbanization and rapid migration of human populations to megalopolis areas set breeding grounds for the spread of zoonotic diseases. As a matter of fact, the leading factors for emerging infections are shanty towns and slums, which have been overcrowded, causing shortage in freshwater supply, sanitation facilities, and proper housing. Prominent examples of infectious diseases that are widely spread among slums include cholera, which has been evidently seen in the slums of Dar es Salaam, Tanzania, an area classified as a megacity given its dense population and low income. In addition, overcrowded housing in the slums of megalopolis regions, such as Dhaka City in Bangladesh, is also contributing to breeding infections, such as tuberculosis. In fact, the prevalence of such disease in Dhaka is twice and four times higher than the national average and overall urbanized standards, respectively. Parasitic infections, on the other hand, such as Chagas disease, are highly prevalent in Latin America, where the population is most dense, and the housing is too poor to prevent vector proliferation. Moreover, heavily urbanized environments, such as New York City, have been evidently rat favorable. New York City, specifically, is regarded as one of the biggest rat populations in America. The constant contact between humans and rats can potentially lead to the risk of zoonoses transmission. Although leptospirosis, a bacterial infection transmitted by rodents such as rats, was initially considered a rural infection, its incidence is growing in urban areas. Additionally, Chinese cities have linked the high incidence rates of Seoul hantavirus hemorrhagic fever with increasing rat populations and human–rat interaction [8], although *Puumala orthohantavirus* and *Dobrava hantavirus* infection are annually reported also in Europe [9].

Moreover, the live animal market and the livestock keepers that maintain direct contact with animals (potentially contaminated) represent high risk scenarios where new zoonotic agents can evolve, adapting itself to new hosts or vectors or becoming more transmissible. Despite the accelerated spread of supermarkets in Asia, notably, live animal markets are continually chosen by 77% of fresh food consumers [10]. 

Although individuals of all ages can be potentially affected by zoonotic diseases, young children and immunocompromised pediatric patients are considered high-risk populations [3].

A long list of pathogens is present in the EID provided by the World Health Organization (WHO), including bacteria, fungi, viruses, and parasites. On this basis, zoonotic diseases can be divided into bacterial zoonoses (e.g., anthrax, salmonellosis, botulism, plague, tuberculosis, leptospirosis, listeriosis, melioidosis, and brucellosis), fungal zoonoses (such as ringworm), viral zoonoses (rabies; HIV; avian influenza; hantavirus; hand, foot, and mouth disease; *Nipah henipavirus*; human coronaviruses; viral hepatitis A; and Ebola), and lastly, parasitic zoonoses (such as toxoplasmosis, trematodosis, giardiasis, trichinosis, taeniasis/cysticercosis, and echinococcosis) [11]. Among the pathogens, there are also vector-borne zoonoses, transmitted by mosquitoes or ticks, such as the *chikungunya virus*, *Crimean–Congo hemorrhagic fever orthonairovirus*, dengue virus, Zika virus, Japanese encephalitis virus, Rift Valley fever virus, and bacteria as the *borrelia* causing Lyme diseases [12]. Among emerging pathogens, 72% are of wildlife origin, and zoonotic pathogens are twice more likely than nonzoonotic ones to be correlated with emerging infectious diseases [11,13].

A particular group of zoonoses is the neglected zoonotic disease (NZD), conditions that are associated with poverty in developing countries (e.g., anthrax, brucellosis, leptospirosis, leishmaniasis, Q fever, toxoplasmosis, anaplasmosis, foodborne trematodes, ehrlichiosis, bartonella, Chagas disease, and toxocariasis. Nevertheless, these diseases are also found worldwide due to emigrations, travel, food, and animal markets [14].

The issues related to zoonosis are multiple, and the complete eradication of such infections is not feasible, although prevention and control measures could help in limiting the spread [11]. Indeed, unique agents, such as smallpox, were completely eliminated by vaccination campaigns [15]. However, there remains a lack of efficient eradication methods for other agents, especially, as they are harbored by multiple wildlife animal reservoirs [11]. Of note, zoonosis and the related health issues are not limited to tropical environments, impoverished areas, and megalopolis; in fact, the European report on zoonosis showed a similar trend of infection extending from 2016 to 2020, indicating that the challenge of zoonosis does not appear to be any less in developed countries either. The most diagnosed zoonosis are (in order from the most to the least frequent): campylobacteriosis, salmonellosis, Shiga-toxin-producing *Escherichia coli* (STEC) infections, yersiniosis, listeriosis, tularemia, Q fever, echinococcosis, Nile virus infection, brucellosis, tuberculosis (*Mycobacterium Bovis, Mycobacterium caprae*), trichinellosis, and rabies [16]. Severe acute respiratory syndrome coronavirus 2 (SARS-CoV-2) has also been inserted in the list of zoonotic viruses. The WHO proposes that the most probable way of virus introduction in the human population should have occurred through an intermediate host from bat reservoirs, even though the identification of this animal is still speculative [1].

A very recent outbreak of monkeypox virus infection has been reported in regions outside Central and Western Africa, where the virus is endemic. Commonly, monkeypox virus disease is found in individuals travelling from African regions, while currently, a great part of the confirmed cases are not travelers from endemic areas, so WHO suggests “undetected transmission for some unknown duration of time followed by recent amplifier events” [17].

With that being said, research must advance at levels that prevent zoonotic diseases from imposing such a threat on public health due to the high prevalence of infectious diseases caused by multi-host pathogens [18]. Today’s society is far from completely eradicating such infections, considering the limited understanding of animal determinants in zoonoses and their causes of emergence. Therefore, efforts must be doubled in examining the spread and persistence of zoonotic diseases instead of merely studying their pathogenicity and possible clinical interventions. The identification, management, and prevention should all be of equal importance and the central focus of research and public health communities [19].

Current preventive and control measures against zoonoses include quarantine, immunization, environmental hygiene, early diagnosis and mass treatment, chemoprophylaxis, education, and slaughter of infected animals, as well as the use of antimicrobial agents.

Quarantine is the restraint of movement or an isolation placed on humans, animals, plants, or foods suspected to carry infectious pathogens or to have been priorly exposed to sources of infectious agents. Mass immunization is performed to minimize the number of susceptible hosts in either human or animal populations, which allows the augmentation of herd immunity, reducing the spread of zoonotic pathogens. Some measures to prevent rabies, for instance, include increasing access to vaccinations for postexposure prophylaxis (PEP), which is not as effective as targeting the primary disease reservoir, canines, through mass vaccinations. Nevertheless, vaccinations for many diseases are still lacking [20]. 

Generally, environmental hygiene control methods against zoonotic diseases involve the implementation of hygienic farm practices in order to improve the sanitary habitats of animals. Environmental hygiene must be well taken care of since it limits the movement of mechanical vectors, such as ticks, mosquitoes, flies, and lice, as well as prevents the transmission of meat-borne pathogens.

Finally, mass treatment is carried out in affected animal populations in cases of emergency or highly risky prevalence of zoonoses. Such treatment could include the use of coccidiostats in drinking water and the incorporation of anthelmintics in salt feeds or licks, which may be given curatively or prophylactically [21].

Several antimicrobial agents are currently available on the market. Antibiotics against bacterial agents are widely known molecules used to fight bacterial infections. A short—no exhaustive—list of the most frequently employed ones includes aminoglycosides, tetracyclines, macrolides (all from *Actinomycetes*), carbapenems, penicillins, cephalosporins (all from fungi), sulfonamides, and fluoroquinolones (all synthetic) [22]. However, the irrational use of these molecules has led to antibiotic resistance. Therefore, the search for novel antibacterial therapies has engaged the effort of the scientific community, and different alternatives have been described as metallic-based nanoparticles, employing, for example, Ag, Au, ZnO, Cu, Se, TiO_2_, NiO [23]; bacteriophage-based treatments [24]; blue laser therapy; and photodynamic approaches [25,26].

To fight fungal infections, polyenes (i.e., amphotericin B), azoles, allylamines, pyrimidines, and echinocandins are commonly exploited. Unfortunately, also in the case of fungi, therapy-resistant strains have been risen, giving a cause of concern for human health [27]. New antifungal drugs are currently under development, such as fosmanogepix, ibrexafungerp, olorofim, opelconazole, and rezafungin [28].

Viruses are the most challenging microorganism group. Indeed, the higher mutation rate requires continuous vigilance of the strains and the evolvement of drugs to target the viral modifications. Moreover, viruses are difficult to disrupt during their viral cycle inside the host cells. For these characteristics, the health agencies invest efforts in vaccination campaigns as preventive measures against viral infections [29].

## 2. Increasing Interest in New Bioactive Antimicrobial Peptides

During the COVID-19 pandemic, the search for antimicrobial bioactive compounds assumed greater proportions compared with prepandemic periods. New biomolecules can potentially act as important weapons against infectious diseases, while their variability offers an arsenal of possibilities still little explored. Among the biomolecules of emerging scientific interest are antimicrobial peptides (AMPs) [30,31].

AMPs can be considered a new class of therapeutic agents that tackle the challenge of pathogen invasion, having several properties that make them particularly attractive, such as their small size, fast activity, and low chance of resistance development by the pathogenic targets [32,33,34,35,36,37]. They are evolutionarily ancestral molecules that evolved in living organisms over 2.6 billion years ago [37] that played a fundamental role in the evolutionary success of multicellular organisms. Such molecules remain effective weapons in organism defense against a wide variety of pathogens, including bacteria, fungi, viruses, and protozoa [33,38,39,40,41]. AMPs are small multifunctional peptides, part of the innate immunity produced by both complex organisms (eukaryotes, such as humans, animals, plants, and fungi) and prokaryotic microorganisms (bacteria) [37]. In humans and mammals, AMPs act as effectors of innate immunity on skin and mucosal surfaces [42]. Most AMPs are cationic, although also anionic peptides have been described. Cationic AMPs mainly target the microbial membrane, while anionic ones generally present intracellular targets (e.g., ribosomes) [43].

Intriguingly, based on the Antimicrobial Peptide Database [44], 3324 natural antimicrobial peptides have been identified so far and registered in the online database, divided into 2446 from animals (comprehending some synthetic peptides), 391 bacteriocins/peptide antibiotics from bacteria, 346 from plants, 22 from fungi, 8 from protists, and 5 from archaea.

Humans harbor 146 AMPs, while amphibians present the highest diversity, producing 1135 active peptides [44]. Plants, due to lack of mobile immune cells and adaptive immune response and their sessile habit that prevents them from evading adverse environmental situations, have 346 AMPs against pathogens [44].

**Figure 1:**
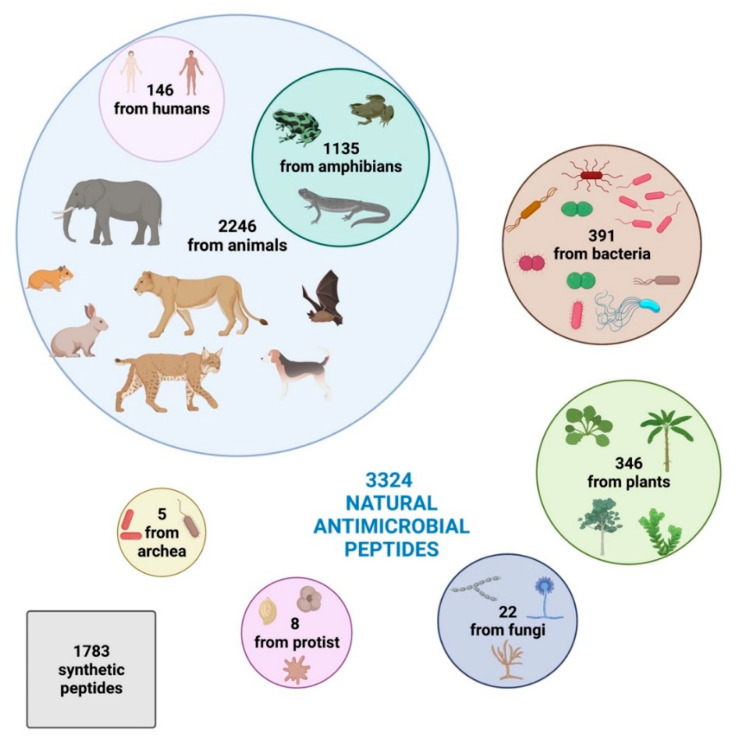
Distribution of AMPs in the six kingdoms according to the Antimicrobial Peptide Database [44] and Data Repository of AntiMicrobial Peptides (DRAMP) database [45].

Moreover, according to the Data Repository of AntiMicrobial Peptides (DRAMP) database [45], 1783 synthetic peptides have been developed and registered (Figure 1). 

Nevertheless, vegetable diversity is still little explored with respect to the broader knowledge regarding human and animal AMPs [32,33,38,39,40,41]. Among plants, the extremophile ones, being well adapted to resist extreme environmental conditions and capable of defending themselves against different pathogenic attacks, are the most interesting, and thus represent a powerful source of defense molecules, producing snakins, heveins, α-hairpinin, and lipid transfer proteins (LTPs) [38,41,46].

AMPs have diverse and complex antimicrobial activities, showing a wide range of antiviral, antibacterial, and antifungal properties and modes of action [36].

An additionally important immunomodulatory activity in humans has been also reported through which endogenous AMPs activate the host’s immune system [36,47], an effect also attributed to when exogenous, synthetic, and vegetable AMPs are delivered. 

The mechanisms of antibacterial action can be divided into membrane and nonmembrane targeting.

The first one is characterized by the formation of membrane pores, leading to the loss of intracellular ions and metabolites, depolarization, osmotic shock, and cell death [48]. It is characteristic of the cationic AMPs. Indeed, the cationic charge allows the interaction between the peptides and plasma membrane components, which results in the accumulation of these molecules on the surfaces. Anionic AMPs can also target the membrane through the exploit of metal ion cofactors, forming cationic salt bridges linked to the negatively charged membrane components of microorganisms [49].

**Figure 2:**
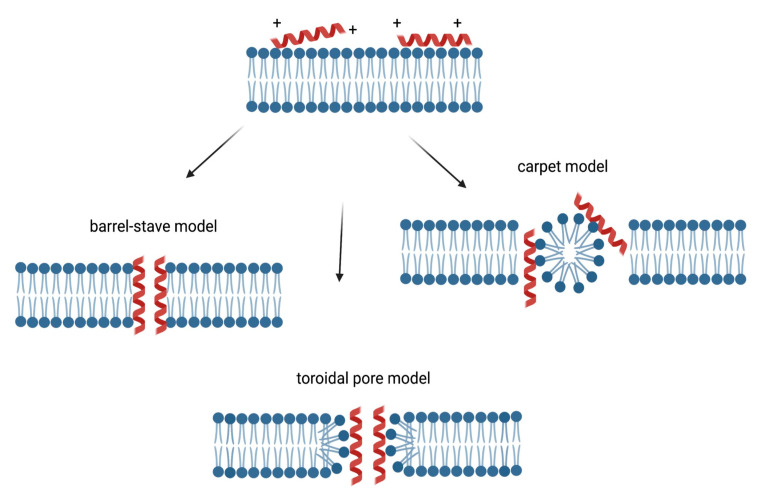
Membrane targeting mechanism of action of cationic AMPs. The cationic charge allows the interaction between the peptides and plasma membrane, resulting in the accumulation of these molecules on the surfaces. In the barrel-stave model, AMPs aggregate and form a hole with the hydrophilic domains in the lumen, while hydrophobic domains come in contact with the lipid bilayer [50]. In the toroidal pore model, AMPs enter perpendicularly the membrane, dragging and bending the lipids as they form a ring hole [50]. In the carpet model, AMPs can act like detergents by locating at the level of the plasma membrane, causing alterations, followed by destruction [51].

Three models of action mechanisms have been proposed for cationic AMPs and are graphically displayed in Figure 2. In the barrel-stave model, AMPs aggregate and form a hole with the hydrophilic domains in the lumen, while hydrophobic domains come in contact with the lipid bilayer. In the toroidal pore model, AMPs enter perpendicularly the membrane, dragging and bending the lipids as they form a ring hole [50]. 

In addition, in the carpet model, AMPs can act like detergents by locating at the level of the plasma membrane, causing alterations, followed by destruction [51]. In Figure 2, the membrane targeting mechanisms of action of cationic AMPs are displayed.

In the nonmembrane mechanisms of action, AMPs enter the cells with a direct penetration or endocytosis. Once inside the cells, AMPs may have different targets. AMPs can affect the transcription, translation, and protein folding through the interference with involved effector enzymes or molecules. It has been shown that AMPs, predominantly anionic AMPs, can directly target ribosomes [52] and chaperons [53]. AMPs can also inhibit protease activity, as serine protease, elastase, and chymotrypsin [53], or target and degrade DNA or RNA, inhibiting key enzymes of nucleic acid biosynthesis but also interfering with DNA replication and nucleic acid damage response [54,55,56]. Finally, AMPs (e.g., Histatin 5) can target fungal mitochondria, leading to reactive oxygen species generation and cell death [57].

AMPs’ activity against fungi partially reflects the antibacterial mechanisms of action, through the interaction with membranes and formation of pores, as well as other specific antifungal properties, such as the targeting of the wall components and related synthase enzymes or nucleic acid, causing DNA binding or intercalation [58].

Against parasites, AMPs display similar processes by recognizing anionic phospholipids on the membrane, therefore causing osmotic imbalance and cellular shrinkage. Besides, they can also impact intracellularly producing mitochondria alterations or disruption of lysosome after endocytosis, leading to cell death [59].

Antiviral action mechanisms are different due to the high diversity of pathogenic viruses. AMPs can directly act on the virion particles targeting external viral structural proteins, inhibiting adsorption or penetration, or they can interfere inside the host cells, hindering viral proteases (essential for viral replication) or impeding viral uncoating and release [60].

Besides natural AMPs, synthetic AMPs (SAMPs) have recently emerged as a new exciting possibility of drug discovery. An AMP with affinity for a bacterial, fungal, or viral protein can not only be identified with the aid of computational strategies but also be further optimized by peptide engineering techniques to make the binding molecule more specific against a molecular target of a pathogen. Hence, based on the sequence of natural AMPs and with the help of such techniques, it is possible to develop SAMPs.

Based on AMP sequences retrieved from public databases, SAMPs can be easily developed with specific characteristics that achieve the required final effect by employing different approaches (e.g., the selection of the known functional motives, site-direct modification/mutation, or de novo construction methods) [43,61].

In this context, genome mining tools can be exploited. Sequence mining techniques involve the identification of previously uncharacterized biomolecules within the available genomes of sequenced organisms [62]. However, this is not an easy task, especially for biomolecules, such as AMPs. These peptides have low conservation of amino acid residues in their sequence due to host–pathogen selective pressure [41]. In recent years, several researchers have presented different solutions to this problem. It has been discussed that a single technique may not be able to encompass all the possible variations that these molecules may present. Thus, the most appropriate way to perform this search would be using different approaches with different search strategies, including search by pattern, as in the case of regular expression techniques (regex) and hidden Markov models (HMM), besides the traditional local alignments (BLAST) [63]. Once these sequences are identified, they can be compared with those of known functions in public databases. These sequence comparison tools are freely available on different websites. Furthermore, these sequences can be used in peptide design techniques to improve their predicted activity and reduce the cytotoxicity that these peptides may present [41]. Online tools like antiSMASH (available at https://antismash.secondarymetabolites.org/#!/start, accessed on 29 June 2022), an automatic miner that uses an algorithm similar to MultiGeneBlast, can be used to identify biomolecule clusters from a database.

As a result, studying these biomolecules has gained great significance due to the emergence of resistant pathogens and other potential pandemic microorganisms. The disorderly use of antimicrobial drugs increases the demand for such peptides because of their inhibitory ability against a broad spectrum of pathogens, which raises the possibility of the appearance of a new generation of antibiotics [64].

## 3. Evidence of the Potentiality of Synthetic Antimicrobial Peptides against Zoonoses

Despite undeniable scientific advances, pathogenic diseases continue to raise significant concerns due to their evolutionary and adaptive capacity. Consequently, the search for novel bioactive biomolecules using different mechanisms of antimicrobial action has been revitalized, gaining more attention given the current pandemic scenario.

SAMPs have been previously exploited in the challenge against zoonosis. In the following sections, some examples of the already proven efficacy of SAMPs are presented.

### 3.1. Viral Zoonosis 

SAMPs can inhibit or interfere with different mechanisms of infection or replication of SARS-CoV-2, the betacoronavirus responsible for COVID-19, affecting primarily the respiratory system [65].

The θ-defensin analog RC101 is effective against SARS-CoV-2 pseudoparticles in vitro when the treatment was performed prior to virus inoculation. Moreover, RC101 possesses an antiviral effect against replication-competent SARS-CoV-2. These peptides partially act when administered to cells prior to pseudovirus infection but not postinfection. Further assays reveal that they mainly affect viral fusion and entry, possibly with a direct impact on the virions, but not the binding of the virus to ACE2 [66].

Diamond et al. [67] showed the potential use of peptoids against SARS-CoV-2. Peptoids are sequence-specific N-substituted glycine oligomers with a backbone of amide nitrogens, representing an example of non-natural peptidomimetic molecules. These modifications render peptoids resistant to host protease, improving also their stability and bioavailability. MXB-4 and MXB-9 peptoids show antiviral activity against SARS-CoV-2 when incubating with the virions for 1 h, probably with a membrane-disruptive mechanism of action.

Several recent studies were also conducted in silico by performing molecular docking.

A good example was presented by Ling et al. [68], who designed SAMPs by molecular dynamics simulation, aiming to bind in the spike protein fusion region of SARS-CoV-2 (HR1 and HR2 regions) with the peptide derived from the spike itself. The binding energy of the predicted HR-2-derived peptide was more substantial than the natural stage of the fusion core, suggesting that the predicted antiviral peptide may competitively bind to HR1 to prevent the formation of the fusion core.

In a similar approach, Mahmud et al. [69] used computational predictive tools to select four potential candidates (out of an AMP database) with the ability to inhibit the main protease of SARS-CoV-2 (Mpro). The selected peptides (circulin A from *Chassalia parviflora*, piscidin 4 from *Morone chrysops* x/ *Morone saxatilis*, neutrophil defensin 1 from *Pan troglodytes* (chimpanzee), and corticostatin-3 from *Oryctolagus cuniculus* (rabbit)) bind to the active cavity of Mpro, in addition to the substrate binding sites (domain 2 and domain 3). The authors suggest that these AVPs may inhibit the Mpro of SARS-CoV-2 even though more laboratory experiments are needed to confirm such results.

An analysis of more than 50 peptides was performed computationally in order to verify the binding capacity to the receptor-binding domain (RBD) of the S protein of SARS-CoV-2. Of these, 15 peptides showed a higher affinity for the human ACE2 receptor. Two of the most promising candidates (S2P25 and S2P26) blocked the entry of SARS-CoV-2 by molecular dynamics simulation tests. Such findings may facilitate the rational design of peptides able to selectively inhibit the action of the SARS-CoV-2 S protein [70].

Zika virus (ZIKV) causes the ZIKV fever, a relatively mild illness (infected individuals present fever, joint pain, headache, cutaneous rash), but in a minority of the cases, it can result in the Guillain–Barré syndrome, a neuropathy affecting the peripheral nervous system and producing muscle weakness, which can be vertically transmitted, producing microcephaly in fetuses [71]. 

He et al. [72] tested several peptides derived mainly from human and bovine cathelicidins to determine their anti-ZIKV activity. Two AVPs (GF-17 and BMAP-18) stood out by showing strong in vitro activities against infected cells. These peptides effectively inhibited ZIKV, regardless of whether they were added before or after infection, suggesting that their mechanism of action is through direct virus inactivation and via the interferon pathway.

Indeed, cathelicidin derivatives have gained prominence in the search for an anti-ZIKV peptide. Xing et al. [73] designed an analog peptide from a snake venom, cathelicidin-30 (ZY13), capable of inhibiting ZIKV in vitro and in vivo. Studies regarding the mechanism of action revealed that ZY13 could directly inactivate ZIKV and reduce the production of infectious virions, in addition to strengthening the host’s antiviral immunity through the cytokine signaling protein suppressor pathway. The authors point to ZY13 as a promising anti-ZIKV drug candidate, highlighting the potential of animal venom peptides as models for developing AVPs.

The dengue virus (DENV) is a significant and growing public health problem worldwide. According to a WHO survey, approximately 400 million people are infected annually. Although most of the infected individuals are asymptomatic, approximately a quarter of them develop a wide range of clinical manifestations with unclear pathogenic mechanisms [74]. To date, there is no specific antiviral drug available to treat DENV infection. In turn, the licensed dengue vaccine (CYD-TDV or Dengvaxia^®^) presents some limitations for use, especially for children under 9 years of age [75].

HS-1, a synthetic peptide derived from anuran *Hypsiboas semilineatus* was tested in vitro against DENV serotypes 2 and 3, showing that HS-1 was active against DENV when the peptide and the viruses were cotreated and then inoculated to the cells, as well as when the binding and internalization assay was performed, while there was no impact when HS-1 was added post-infection or the cells were treated prior to viral inoculation. Interestingly, the cotreatment induced destabilization of the viral envelope, and it was also efficacious in vivo, when the treated viruses were used to infect mice [76].

Reported targets of peptide inhibitors against DENV infection generally include viral structural proteins, such as C, prM, and E, as well as viral NS2B-NS3 protease and NS5 methyltransferase [77]. For instance, Tambunan and Alamud [77] designed seven peptides against the NS2B-NS3 protease of dengue virus type 2 (DENV-2) to block active sites of viral proteins or to mimic specific regions of viral proteins as competitive inhibitors of viral entry and viral replication. In addition to the regions cited as promising targets, Songprakhon et al. [78] studied the NS1 protease as a novel peptide target to inhibit DENV. AVPs exhibited varying degrees of inhibition towards different DENV serotypes. Given the promising results, the authors suggest that these peptides could be the start of developing an inhibitor-based project for the multifunctional NS1 protein.

Another currently critical viral disease involves the outbreak of influenza A H3N2. Influenza viruses figure as an example of viruses whose evolution is highly associated with their ability to establish and spread in different host species. They have most likely originated from aquatic birds. Their evolution and ecology have been associated with various native reservoirs, from wild birds to poultry and to various mammalian host species [79]. 

H3N2 is currently spread in different world regions. Between July and November 2020, an influenza A (H3N2) epidemic occurred in Cambodia and other neighboring countries in the Greater Mekong subregion of Southeast Asia [80]. However, the virus mutated again in Australia this year (Darwin variant), enough to increase emergency room and hospital admissions. H3N2 was recently spread in Brazil together with an outbreak of the Omicron variant of SARS-CoV-2, resulting in many hospitalizations and deaths [81].

The influenza A virus presents a high mutation capacity, requiring a new vaccine for each new variant.

Studies by Li et al. [82] showed that the fish-skin-derived SAMP peptide P acts as a potential natural inhibitor of influenza A neuraminidase in MDCK cells in the early stage of the infectious cycle. Molecular docking simulation indicated that the SAMP could directly bind neuraminidase, being a competitive inhibitor. In the in vitro assays, peptide P was able to protect the cells from infection and to reduce virus replication. The major effect was obtained when SAMP was added prior to viral inoculation, while a minor impact was observed when it was supplemented during or post-viral absorption. These data suggest that peptide P may target the cell surface and prevent virus binding to the host cell, and the hemagglutination (HA) assay confirmed these results.

### 3.2. Bacterial Zoonosis

SAMPs can also be used as a valid strategy for targeting bacterial zoonoses.

Foodborne zoonotic infections impose a great threat to human consumers. Although in Europe in 2020, 120,946 notified cases of campylobacteriosis, 52,702 of salmonellosis, 4446 of STEC, 5668 of yersiniosis, and 1876 of listeriosis were reported, the real numbers may be, indeed, higher [16]

Campylobacteriosis, commonly transmitted by contaminated avian meat, is caused by *Campylobacter* species (mainly *C. jejuni*, *C. coli*, and *C. lari*), and is characterized by diarrhea, abdominal pain, nausea, and fever [83].

Talukdar et al. showed that puroindoline A (PinA) peptides derived from puroindolines (*Triticum aestivum*) displayed a strong antibacterial activity against *Campylobacter jejuni*, inhibiting its growth by disrupting its cellular membrane, while also blocking biofilm formation [84].

Salmonellosis, typically, provokes stomachache and diarrhea, but other symptoms include vomiting, nausea, fever, and muscular/articular pain. The transmission occurs through the ingestion of eggs and pig meat as well as cattle and dairy products. Despite the existence of different *Salmonella* serotypes, the nontyphoid ones (*S. Typhimurium, S. enteritidis, S. newport,* and *S. Heidelberg*) are those responsible for food contamination [85].

Cap-18 (from rabbit neutrophils, analog to the human LL-37) is an AMP with activity against *Salmonella typhimurium* in vitro. A library of 696 Cap-18 derivatives (each with a single amino acid substitution of the original peptide sequence) was screened against *Salmonella typhimurium*, showing that about 82% of the molecules tested presented the same activity as the original AMP, but 5 of them displayed a higher effect. The screening resulted in identifying the key amino acid residues for the antimicrobial activity of Cap-18. Moreover, the authors assayed the library against the beneficial *Lactococcus lactis* to determine the species specificity of the peptides [86].

Talukdar et al. [84] showed that puroindoline A (PinA) peptides derived from puroindolines (*Triticum aestivum*) are effective in counteracting the growth and biofilm formation of *Salmonella enterica serovar Typhimurium.*

*Yersinia enterocolitica* is the causative agent of yersiniosis. The stability of *Yersinia enterocolitica* in cool conditions (4 °C) and its ability to produce thermostable toxins are problematic features for humans consuming contaminated food. The classical manifestations of yersiniosis are fever, stomachache, and bloody diarrhea, mimicking appendicitis. The infection can be transmitted through pork, milk and dairy derivatives, plants, seafood, and water ingestion [85].

Sijbrandij et al. [87] tested SAMPs derived from bovine lactoferrin against *Yersinia enterocolitica,* finding a strong bactericidal effect due to permeabilization and depolarization of the bacterial membrane. Moreover, SAMPs inhibited also the bacterial host cell invasion (in HeLa cells) by inducing inflammatory mediators released by the HeLa cells [88]

*Listeria monocytogenes* causes listeriosis and is a Gram-positive bacterium able to withstand unfavorable conditions, such as 0–45 °C of temperature and a 4.4–9.4 pH range. *Listeria monocytogenes* are intracellular bacteria that penetrate intestinal cells, producing gastrointestinal symptoms (vomiting, stomachache, diarrhea, weariness), in addition to the cells of the spleen, liver, brain, and heart. Most cases required hospitalization with a mortality rate of 20–30% [85]. Although this pathogen is commonly found in the environment, it is, in fact, also present in domestic animals (cattle goats, horses, poultry, fishes) through which it is transmitted to humans by alimentation.

Talukdar et al. [84] found that Puroindoline A (PinA) is able to disrupt the membranes of *Listeria monocytogenes*, therefore blocking their expansion and the biofilm development.

Shiga-toxin-producing *Escherichia coli* (STEC) is a bacterium carrying the genes for the expression of Shiga toxin types 1 (Stx1) and 2 (Stx2). STEC infection causes bloody diarrhea, nausea, headache, vomiting, and abdominal cramping, with a fatality rate of 5% in children and a risk of 15% of developing hemolytic uremic syndrome and life-threatening renal failure. Transmission mainly occurs through the consumption of beef, water, and milk [89].

Lino et al. [90] showcased the antimicrobial effect of the d-amino acid hexapeptide WRWYCR against STEC. The peptide inhibited bacterial DNA repair through the strong binding to the Holliday junction intermediates blocking the resolution. Acidic stress decremented the STEC survival; nevertheless, if a pretreatment with the peptide was performed, a major reduction was achieved. The most effective protocol, where no detectable survival was measured, was formed by room temperature treatment of the bacteria with the peptide, followed by acidic treatment at 37 °C, possibly suggesting a clinical pre-ingestion approach combined with the subsequent exposure to the gastric acidic environment. Finally, the authors detected no increase in Shiga toxin production in the acidic condition.

### 3.3. Fungal zoonosis

Fungal infections have gained notoriety as an alarming problem in recent decades given the evolution of intrinsic resistance to current antifungals [91]. For *Cryptococcus neoformans*, a human fungal pathogen that mainly affects immunocompromised individuals, reports of resistance to usual therapeutics are limited [92]. Data from the Leading International Fungal Education (LIFE) website in 2017 (updated in 2018) estimate around 223,100 annual cases of cryptococcal meningitis in the immunocompromised, with a mortality rate of 10% and greater than 70% in the US and Africa, respectively, leading to approximately 181,000 deaths per year [93,94].

In this context, intensive research has been carried out using antifungal peptides (AFPs) against a diversity of fungi due to their efficacy and high selectivity [95]. Zhang et al. [96] tested an AFP, known as SP1 and derived from *Saccharomyces cerevisiae,* that showed potent activity against *C. neoformans* and *C. gattii*. Unlike most AFPs that form pores in the pathogen’s cell membrane, SP1 interacts with the pathogen’s membrane ergosterol and enters the vacuole, possibly through natural membrane traffic, causing calcium ion homeostasis imbalance, increased reactive oxygen, exposure to phosphatidylserine, and nuclear fragmentation. Finally, the authors suggest that SP1 has the potential to be developed as a treatment option for cryptococcosis. 

Specht et al. [97], in a recent publication, demonstrated the effects of a potential peptide vaccine against cryptococcosis (*C*. *neoformans*). This peptide with 32 amino acids was able to strongly bind to the major histocompatibility complex class II (MHC II) H2-IAd allele in BALB/c mice. Therefore, the authors conclude that peptide-based vaccines containing a single peptide can protect mice against cryptococcosis. However, given the diversity of human MHC II alleles, a single-peptide-based vaccine would be challenging for human use and would likely require multiple peptide sequences.

Another disease that has gained importance in recent years due to its worldwide prevalence, distribution, and epidemiology is sporotrichosis (caused by species of fungi of the genus *Sporothrix*) [98,99]. This disease has different clinical manifestations (cutaneous, lymphocutaneous, and disseminated) and could also progress to a systemic infection [100].

Sporotrichosis is more prevalent in tropical and subtropical countries. Despite this, it has been reported in the United States, Europe (where cases have been reported intermittently in countries such as France, Italy, Spain, Portugal, the United Kingdom, and Turkey), Asia (China, India, and Japan), Africa (South Africa, Zimbabwe, Nigeria, and Sudan), and Australia [98]. 

Yan et al. [101] selected an AFP (ToAP2) from a database with the aid of bioinformatics tools and constructed three more peptides (ToAP2A, ToAP2C, and ToAP2D) using peptide design techniques. The three derived AFPs inhibited the growth of *Sporothrix globosa*, among which ToAP2D had the best results, displaying strong antifungal activity, good serum stability, and no acute toxicity. Scanning electron microscopy analysis revealed membrane deformation and rupture as the main mechanism of action. Overall, the authors suggest that ToAP2D is potentially therapeutic against sporotrichosis.

### 3.4. Zoonotic parasites

Toxoplasmosis is caused by *Toxoplasma gondii*, an apicomplexan parasite capable of infecting any nucleated cell in any warm-blooded animal. Cats can harbor *Toxoplasma gondii*, and they can be a source of animal–human transmission. About 2 billion people are infected annually; however, a small percentage of them suffer from the severe form of this disease, which can cause serious eye disease, fatal encephalitis in immunosuppressed individuals, and miscarriage or birth defects in pregnancy. The prevalence of this parasite alludes to it as one of the most harmful zoonotic diseases in the world, which is explained by its neurotropic nature and high morbidity and mortality rates in immunocompromised patients and newborns [102].

De Assis et al. [103] tested the anti-toxoplasma activity of peptides derived from the venom of the yellow scorpion *Tityus serrulatus*. As a result, this study observed that such peptides reduced the replication of tachyzoites in macrophages showing no cytotoxicity. Mice infected with *T. gondii* were treated using the aforementioned peptides, showing a decrease in the number of brain cysts while inducing no hepatotoxicity in the animals tested. Hence, the authors conclude that the data present promising immunomodulatory and antiparasitic activities of these peptides, which could be explored and applied in future therapies to treat toxoplasmosis.

In a similar approach, Liu et al. [104] tested a peptide (XYP1) derived from the venom gland of the spider *Lycosa coelestis*. This peptide exhibited potent anti-toxoplasma activity in vitro and in vivo. XYP1 treatment significantly inhibited the viability, invasion, and proliferation of tachyzoites in human host cells, with no cytotoxicity and increased the survival rate of mice acutely infected with *T. gondii*. Notably, the mechanism of action of XYP1 may be related to tachyzoite membrane disruption. In conclusion, the authors propose the possibility of XYP1 being a promising new drug candidate for the treatment of toxoplasmosis.

Hookworm is an intestinal parasite that infects nearly 230 million people, with another 5.1 billion at risk, especially in poverty-stricken tropical and subtropical regions [105]. Chronic hookworm infection can induce iron deficiency anemia, with children and women of childbearing age being the most vulnerable. Currently, control efforts rely on the mass administration of drugs that treat established infections but do not prevent reinfection [106].

In search of new anthelmintics, SAMPs hold great promise, especially the cyclotide family, plant cyclic peptides with approximately 30 aa [41], which have already been presented with anthelmintic activity [107]. Colgrave et al. [108] demonstrated the in vitro anthelmintic activity of three cyclotides—kalata B1, kalata B6, and cycloviolacin O14—on the viability of the larval and adult stages of the canine hookworm *Ancylostoma caninum* and only the larval stage of the human hookworm *Necator americanus*. The authors conclude by reinforcing the promising activity of cyclotides as novel anthelmintics.

Human taeniasis is an infectious disease caused by the ingesting of the larval stage of metacestode, the cysticerci of *Taenia saginata* in beef or *Taenia solium* in pork, which draws great attention for its ability to cause neurocysticercosis, one of the main causes of neurological system morbidity worldwide [109]. The taeniasis/cysticercosis complex is included in the list of neglected tropical zoonoses of the World Health Organization and that of the Food and Agriculture Organization [110]. This disease is more prevalent in the regions of Asia, Africa, and Latin America, where the disease remains endemic [111].

Landa et al. [112] tested the ability of two AMPs, temporin A (TA, from frog *Rana temporaria*) and Iseganan IB-367 (IB-367, a synthetic analog of porcine protegrin) to damage *T. crassiceps cysticerci in vitro*. These peptides caused cysticerci shrinkage, loss of motility, formation of macrovesicles in the tegument, and a decrease in evagination properties. In addition, peptides administered to cysticercotic mice 1 month after infection in a single intraperitoneal dose reduced the parasite load by 25% to 50%. The authors suggest that these findings may contribute to the design of new drugs to prevent and treat such diseases.

Leishmaniasis is a zoonotic disease caused by sand flies infected with an obligate intracellular protozoan parasite (family *Trypanosomatidae*), which presents itself in three different forms: cutaneous, mucocutaneous, and visceral [113]. The clinical manifestation of this disease varies according to the parasite species and ranges from physical disfigurement to death if left untreated. Despite being first discovered in India, the parasite has been located in several other countries around the world as well. As of 2018, according to the World Health Organization, 94% of the total new cases occurred in seven countries: Brazil, India, Kenya, Somalia, South Sudan, Ethiopia, and Sudan. The disease is endemic in 88 countries, 72 classified as developing countries [114].

Peptide-based drugs are currently being used to develop innovative therapies for various health conditions, including tropical diseases, such as leishmaniasis [115].

In this context, Kumar et al. [116] tested tachyplesin peptide (from the Japanese horseshoe crab *Tachypleus tridentatus*) against *Leishmania donovani*. This peptide was established to be active against both forms of the parasite, showing no toxicity to host cells at the concentration used while exhibiting a mode of action that destabilizes the membrane of the protozoan, thus hindering the development of resistance of this parasite. The authors conclude by emphasizing the importance of further analyzing the peptide/parasite interaction and stating that the anti-leishmanial property of tachyplesin makes it appealing as a future drug in leishmaniasis treatment.

Cao et al. [117] examined a peptide of only four amino acids (KDEL, based on the *Pseudomonas aeruginosa* exotoxin PE) against the promastigote and amastigote of *Leishmania tarentolae*. The results illustrated the dose-dependent activity of the peptide against this protozoan. In addition, it was possible to observe that the mode of action of this peptide was directly linked to the ability to disrupt the integrity of the parasite’s surface membrane and thus cause cellular apoptosis. Despite having only four amino acids, KDEL has shown therapeutic potential as a new antileishmanial drug.

In Table 1, a summary of the candidate SAMPs against zoonotic agents is displayed.

## 4. Clinical Development of SAMPs

The latest report by Transparency Market Research showed that the global AMPs’ pharmaceutical market has been growing at an annual rate of approximately 8%, and it is estimated to reach a value of USD 50 billion by 2027, according to the latest report by Transparency Market Research [118].

Nevertheless, according to the Data Repository of AntiMicrobial Peptides (DRAMP) database [45], at the moment, only 77 peptides have been developed by pharmaceutical companies. A peptide, DP178 (T20, enfuvirtide and Fuzeon), an HIV-1 membrane fusion inhibitor, was approved as an antiviral by the FDA [45]. Interestingly, the DP178 peptide shares the sequence of the 643–678 residues of the gp41 of HIV-1 (LAI isolate) and inhibits HIV-1-mediated cell–cell fusion and syncytium formation with a 50% inhibitory concentration of 0.38 nM combined with no cytotoxic effect [119].

Moreover, other AMPs in clinical use against bacteria include those against Gram-positive bacteria, such as daptomycin (a cyclic lipopeptide from *Streptomyces roseosporus*), oritavancin, dalbavancin, teicoplanin and telavancin (semisynthetic glycopeptide antibiotics derived from vancomycin), and gramicidins (linear peptides from *Bacillus brevis*), and against Gram-negative bacteria, such as polymyxins (cyclic peptides from *Paenibacillus polymyxa*) [120].

Of note DRAMP contains 22407 entries (natural and synthetic), 16110 of which are patented AMPs, showing the wide gap between the discoveries and design of new peptide sequences, the obtainment of patent (~71% of the total entries), and the clinical development (~0.33% of the total entries). Other 5909 candidate AMPs are also reported, but their antimicrobial action has not yet been assessed.

These data are currently being updated, and the information here presented is only an instantaneous picture of the current peptide landscape (up to 29 July 2022). The number of entries will, in fact, continuously increase. However, these numbers would be helpful to highlight that, besides the antimicrobial properties, tested mostly with in vitro assays, the development of SAMPs as human drugs is a long issue.

As a rule, successful SAMPs should possess clear efficacy and be superior to conventional treatments. Nevertheless, in this evaluation it is not considered that SAMPs have the advantage of inducing less microbial resistance as compared with conventional antibiotics, a characteristic that must not be ignored (reviewed by [121]).

Another pitfall is related to the low specificity of SAMPs towards prokaryotic cells. Indeed, the main target of SAMPs is the lipidic bilayer, the main structure of all types of cells, often toxic also for eukaryotic cells [122]. The slight balance between concentrations with antimicrobial action and a safety profile for human cells should be carefully determined (reviewed by [121]).

A crucial aspect is the stability in human body environments. Indeed, degradation by protease, binding to host proteins, and modifications in the charge conditions are all situations where the SAMPs’ efficiency is reduced and, in extreme cases, lost (reviewed by [121]).

Therefore, improvement strategies are mandatory to increase the stability, bioavailability and biodistribution of SAMPs in the host organism.

An initial bioinformatics approach to retrieve peptide sequences from the database, to modify peptide sequence, characteristics, and dynamic simulation to screen libraries of potential peptide candidates based on their characteristics and their binding/activity, would be the starting point to improve SAMPs at a low cost that is actually employed by big pharmaceutical companies for drug discovery [60].

Chemical modifications can increment the hydrophobicity and decrement net positive charge, leading to cytotoxicity reduction. Moreover, the introduction of D-amino acids, cyclization, amidation, or acetylation at the terminal ends improves the AMPs’ stability (reviewed by [121]).

Other interesting approaches are the design of specifically targeted AMPs (STAMPS), where a target region improves the binding of the AMPs to a specific pathogen feature; then a killing region promotes the kill of the microorganism [123] or the employment of AMPs combined with antibiotics, which promotes a synergism between the two molecules [124]

**Figure 3:**
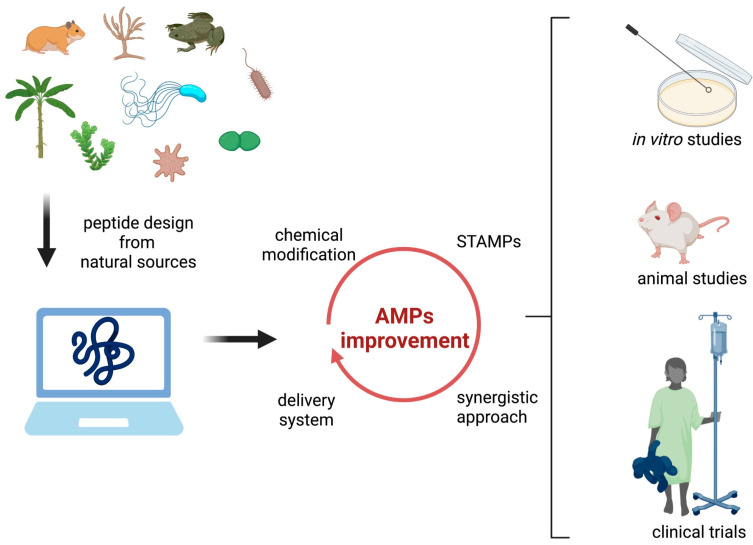
The development of new synthetic AMPs.

Delivery systems can be implemented with the encapsulation of SAMPs in nanoparticles, micelles, and enzymatically controlled vehicles (reviewed by [121]; Figure 3).

## 5. Prospects for Future Development of Therapeutic Antimicrobial Peptides

In the current pandemic scenario, attention to preventing and controlling zoonotic diseases is in continuous growth. Indeed, in both worldwide populations and scientific communities, the awareness of danger associated with the zoonotic pathogens has abruptly arisen [125].

Thus, therapeutic alternatives that can be used to contain the spread of these pathogens have become of fundamental importance to counteract the current microorganisms while also improving the preparedness towards the spring out of new emerging infectious agents [125]. In recent decades, research involving AMPs has gained prominence in the scientific community due to their various reported mechanisms of action: broad-spectrum antimicrobial, anticancer, and anti-inflammatory activities, among others. Since AMPs play a vital role in the initial nonspecific immune response, they comprise an almost inexhaustible source of biologically active molecules with therapeutic potential [41].

Intriguingly, it has been shown that synthetic AMPs are more efficient at a low concentration with respect to their natural counterpart. Indeed, synthetic AMPs can be optimized to enhance their effect and eliminate the motifs responsible for toxicity or instability [126].

Despite the advantages, AMPs present some limitations that have been limited so far by their applicability in real clinical settings, such as the in vivo stability and toxicity caused by the binding of the AMPs to the host cell membrane; not strict correlation between their in vitro and in vivo efficacy, with AMPs being highly affected by the environmental conditions; and the high production cost [127].

To date, the in vitro testing of the SAMPs is limited to a small number of species, and data report the activities of a few SAMPs against the most common pathogens (not all from a zoonotic origin). Therefore, a high-throughput screening of SAMP libraries against a wide range of pathogens would be envisaged to elucidate new potential SAMP applications [128].

Previous studies on the efficacy of antimicrobial peptides, although very promising, are still in their early stages; indeed, further studies and better characterization of their mechanism of action with in vitro and in vivo assays are needed so as to proceed to their clinical application on human beings.

## Figures and Tables

**Table 1:** Summary of the candidate SAMPs against zoonotic agents.

Microorganisms	Target Microorganisms	Peptide and Source	Mechanism of Action	Reference
Viruses	SARS-CoV-2	θ-defensin analog RC101 (human)	Affected viral fusion and entry, possibly with a direct impact on the virions	[66]
		MXB-4 and MXB-9 peptoids	Membrane disruptive	[67]
		HR1 and HR2 (SARS-CoV-2)	Binding of the spike protein	[68]
		Circulin A from *Chassalia parviflora* Piscidin 4 from *Morone chrysops*/*Morone saxatilis* Neutrophil defensin 1 from *Pan troglodytes* (chimpanzee) Corticostatin-3 from *Oryctolagus cuniculus* (rabbit)	Inhibition of Mpro	[69]
		S2P25 and S2P26 (synthetic)	Binding of the RBD spike	[70]
	ZIKA virus	GF-17 (human cathelicidins) BMAP-18 (bovine cathelicidins)	Direct virus inactivation	[72]
		ZY13 (snake venom cathelicidin-30)	Direct virus inactivation	[73]
	Dengue virus	HS-1 (anuran *Hypsiboas semilineatus*)	Block of virus binding and internalization	[76]
		Synthetic peptides targeting	Block active sites of viral proteins (NS2B-NS3 protease) Competitive inhibitors of viral entry and viral replication	[77]
		Synthetic peptides	Inhibition of DENV through targeting NS1 protease	[78]
	Influenza A H3N2	Fish-skin-derived SAMPs	Inhibitor of influenza A neuraminidase	[82]
Bacteria	*Campylobacter*	Puroindoline A (PinA) from puroindolines (*Triticum aestivum*)	Inhibiting bacterial growth by disrupting their cellular membranes while also blocking biofilm formation	[84]
	*Salmonella*	Cap-18 derivatives (from rabbit neutrophils, analog to the human LL-37)	Inhibition of bacterial growth	[86]
		Puroindoline A (PinA) from puroindolines (*Triticum aestivum*)	Inhibiting bacterial growth by disrupting their cellular membranes while also blocking biofilm formation	[84]
	*Yersinia enterocolitica*	Antimicrobial peptides derived from bovine lactoferrin	Bactericidal effect due to permeabilization and depolarization; inhibition of host cell invasion by the bacteria	[87,88]
	*Listeria monocytogenes*	Puroindoline A (PinA) from puroindolines (*Triticum aestivum*)	Inhibiting bacterial growth by disrupting their cellular membranes while also blocking biofilm formation	[84]
	Shiga-toxin-producing *Escherichia coli*	Hexapeptide WRWYCR against STEC	Inhibition of bacterial DNA repair, reducing STEC survival, with no increase in Shiga toxin production in an acidic environment	[90]
Fungi	*Cryptococcus neoformans*	SP1(derived from *Saccharomyces cerevisiae*)	Interaction with the pathogen’s membrane ergosterol and enters the vacuole, causing calcium ion homeostasis imbalance, increased reactive oxygen, exposure to phosphatidylserine, and nuclear fragmentation	[96]
	*Sporothrix*	ToAP2A, ToAP2C, and ToAP2D	Inhibition of the growth membrane deformation and rupture	[101]
Parasites	*Toxoplasma gondii*	Peptides derived from the venom of the yellow scorpion *Tityus serrulatus*	Reduced the replication of tachyzoites	[103]
		Peptide (XYP1) derived from the venom gland of the spider *Lycosa coelestis*	Inhibited the viability, invasion, and proliferation of tachyzoites through membrane disruption	[104]
	*Ancylostoma caninum* *Necator americanus*	Kalata B1, kalata B6, and cycloviolacin O14	Reduction of the viability of larval *(Ancylostoma caninum, Necator americanus)* and adult stages (*Ancylostoma caninum)*	[108]
	*Taenia*	Temporin A (TA, from frog *Rana temporaria*) Iseganan IB-367 (IB-367, a synthetic analog of porcine protegrin	Cysticerci shrinkage, loss of motility, formation of macrovesicles in the tegument, decrease in evagination properties	[112]
	*Leishmania*	KDEL, based on the *Pseudomonas aeruginosa* exotoxin PE	Disruption of the integrity of the parasite’s surface membrane and cellular apoptosis	[117]

## Data Availability

Not applicable.
